# Feasibility of implementing a community cardiovascular health promotion program with paramedics and volunteers in a South Asian population

**DOI:** 10.1186/s12889-020-09728-9

**Published:** 2020-10-27

**Authors:** Gina Agarwal, Manika Bhandari, Melissa Pirrie, Ricardo Angeles, Francine Marzanek

**Affiliations:** 1 Department of Family Medicine, McMaster University, David Braley Health Sciences Centre, 100 Main Street West, Hamilton, ON L8P 1H6 Canada; 2 Department of Health Research Methods, Evidence, and Impact, McMaster University, 1280 Main Street West, Hamilton, ON L8S 4L8 Canada

**Keywords:** Cardiovascular disease, Diabetes, Immigrant, South Asian, Community health, Health promotion, Chronic disease prevention, Community paramedicine

## Abstract

**Background:**

The South Asian population in Canada is growing and has elevated risk of cardiovascular disease and diabetes. This study sought to adapt an evidence-based community risk assessment and health promotion program for a South Asian community with a large proportion of recent immigrants. The aims were to assess the feasibility of implementing this program and also to describe the rates of cardiometabolic risk factors observed in this sample population.

**Methods:**

This was a feasibility study adapting and implementing the Community Paramedicine at Clinic (CP@clinic) program for a South Asian population in an urban Canadian community for 14 months. CP@clinic is a free, drop-in chronic disease prevention and health promotion program implemented by paramedics who provide health assessments, health education, referrals and reports to family doctors. All adults attending the recreation centre and temple where CP@clinic was implemented were eligible. Volunteers provided Hindi, Punjabi and Urdu translation. The primary outcome of feasibility was evaluated using quantitative process measures and a qualitative key informant interview. For the secondary outcome of cardiometabolic risk factor, data were collected through the CP@clinic program risk assessments and descriptively analyzed.

**Results:**

There were 26 CP@clinic sessions held and 71 participants, predominantly male (56.3–84.6%) and South Asian (87.3–92.3%). There was limited participation at the recreation centre (*n* = 19) but CP@clinic was well-attended when relocated to the local Sikh temple (*n* = 52). Having the volunteer translators was critical to the paramedics being able to collect the full risk factor data and there were some challenges with ensuring enough volunteers were available to staff each session; as a result, there were missing risk factor data for many participants. In the 26 participants with complete or almost complete risk factor data, 46.5% had elevated BP, 42.3% had moderate/high risk of developing diabetes, and 65.4% had an indicator of cardiometabolic disease.

**Conclusion:**

Implementing CP@clinic in places of worship is a feasible approach to adapting the program for the South Asian population, however having a funded translator in addition to the volunteers would improve the program. Also, there is substantial opportunity for addressing cardiometabolic risk factors in this population using CP@clinic.

**Supplementary Information:**

The online version contains supplementary material available at 10.1186/s12889-020-09728-9.

## Background

South Asians, defined as people originating from Pakistan, India, Bangladesh, Nepal or Sri Lanka, represent one of the largest ethnic visible minority groups in Canada contributing to approximately 4.8% of the total Canadian population [1]. South Asians include individuals with diverse cultural and religious backgrounds. According to Statistics Canada, the number of people of South Asian origin in Canada is growing considerably faster than the overall population and is expected to grow three-fold by 2031 [1, 2].

Evidence suggests that significant disparities in rates of cardiometabolic conditions and health behaviours persist among various ethnic groups in Canada [3]. South Asian communities face an intersection of biological and social determinants of health that places them at a higher risk of various cardiometabolic conditions [1, 3]. Individuals of South Asian descent generally have an elevated risk of developing heart disease, diabetes and other cardiometabolic conditions at an earlier age as compared to the general population, which creates a higher burden on the public health system [3, 4]. Therefore, it is very crucial to target this population with health promotion and disease prevention initiatives.

Compared with general public health education as a health promotion strategy, interventions that assess individual risk, create a supportive environment for health, and strengthen community action, result in better outcomes [5, 6]. Considering the multiple socio-cultural barriers faced by new immigrants transitioning to Canada, health promotion programs that are culturally appropriate and accessible are needed [6]. Religious institutions and other faith organizations are becoming increasingly popular settings in which to conduct health promotion initiatives [6, 7]. Since many South Asian communities identify religion as a social support mechanism, interventions targeting these communities in religious institutions such as temples may be an important pathway for reducing the risk of cardiometabolic conditions in this high-risk population [8]. Given the paucity of scientific evidence on the effectiveness of community-based health assessment programs targeting South Asian populations in Canada, this study is one of the first to implement and test the feasibility of a culturally-salient, evidence-based health promotion and disease prevention initiative in a religious setting (a local Sikh temple) to improve cardiometabolic risk factors among South Asian immigrants in Canada.

South Asian immigrants may therefore be considered a marginalized population. Community Paramedicine at Clinic (CP@clinic) is a program that has previously been effective in other marginalized populations. It is a risk assessment, chronic disease prevention, and health promotion program that was developed for older adults in subsidized housing. The program is implemented by a community paramedic and has been tested in a cluster randomized trial. The results of the trial showed that CP@clinic decreased EMS calls, improved quality of life, and decreased chronic disease risk factors [9]. Paramedics have been shown to be able to develop trusting relationships with marginalized populations in CP@clinic [10]. Also, the intervention is easy to implement in community settings and requires minimal resources which may therefore be appropriate to be implemented in South Asian community settings.

The primary objective of this study was to assess the feasibility of a weekly or bi-weekly CP@clinic program targeting the South Asian population in the Riverdale area, which is home to Hamilton’s largest proportion of new South Asian immigrants [11]. Specifically, whether the key components of the CP@clinic program (e.g. paramedic-led sessions, risk assessments, referrals, reports to primary care) can be feasibly implemented in a South Asian community setting. The secondary objective of the study was to describe cardiometabolic risk factors observed in this high-risk population to inform a future full-scale study and health promotion and disease prevention initiatives.

## Methods

### Study design and setting

This was a feasibility study of the CP@clinic program held in a South Asian community setting. We used process measures and data collected through assessments as part of the CP@clinic program and analyzed these data at the end of the program [9]. In addition, qualitative key informant interviews were conducted to help support the quantitative process evaluation and gather information on how to improve the program.

### Intervention

The CP@clinic program is an evidence-based program that was thoroughly evaluated in a community cluster randomized controlled trial [9]. The key components of the program are: it uses evidence-based tools (validated scales, clinic grade devices) and procedures (physical assessments following practice guidelines), it uses the CP@clinic database with built-in algorithms to guide trained paramedics in initial and follow-up visits, tailored referrals are made to local community resources, and the assessment findings are sent to the participant’s primary healthcare provider to close the loop. In this weekly one-on-one drop-in program, paramedics provide health assessments (diabetes and falls risk, blood pressure [BP], and cardiometabolic risk factors), make referrals to community-based resources, and engage participants in healthy lifestyle conversations. The program also aims to identify patients at high risk of certain cardiometabolic diseases and to ensure timely and regular communication of participants’ health information with their family physician [9]. Participants are encouraged to attend follow-up visits to monitor changes in their risk factors and check if the referrals and advice the paramedics made were followed. A detailed description of paramedic assessments can be found in the published protocol for the CP@clinic randomized controlled trial [9].

The current study was not one of the main trial sites, but was a separate feasibility study using the same assessment tools and community paramedic implementation process. Research ethical approval for this study was granted from Hamilton Integrated Research Ethics Board (Study #: 14–645, September 16, 2014). In this study, the CP@clinic program was being adapted to a South Asian population residing in the Riverdale area of Hamilton, one of Canada’s largest communities of recent immigrants where 51% of residents are foreign-born and 16% are recent immigrants [12].

### Setting

The original program was implemented in subsidized housing buildings where participants were primarily white. This current program was initially implemented in a local community recreation centre. However after 1 month, the program was moved to a local Sikh Temple (Gurdwara Shaheedgarh Sahib Hamilton) to encourage participation from the targeted population. The study was conducted over a period of 14 months (October 2016 to November 2017). In contrast to the original study where only community paramedics delivered CP@clinic, student volunteers assisted the community paramedics. The student volunteers had completed an online and in-person CP@clinic training program [9] which covered the whole program, aspects of obtaining informed consent, and privacy and confidentiality of health information. They held 3-h weekly drop-in sessions every Sunday either in a room at the recreation centre or in the common library located at the temple. The student volunteers (Health Sciences and Pre-Medical students), who were able to speak in Hindi, Punjabi and Urdu, also acted as translators for individuals who did not speak or understand English, assisting the community paramedics in obtaining consent and completing the risk assessments. It was important to have in person translation of the questionnaires since some participants were unable to read.

### Participants

The participants consisted of adults visiting the local community recreation center or the local Sikh Temple. Since the study population was predominantly North Indian, speaking Hindi, Punjabi and Urdu, the study was advertised and participants were invited through information displayed on posters and flyers translated in all three languages. Posters and flyers were also placed in the local South Asian grocery stores, and on the Immigrant Workers Association noticeboard. Flyers were placed in the mailboxes of houses in streets surrounding the recreation centre and posters in the foyers and laundry rooms of the many large housing buildings around the centre. Trained student volunteers assisted with participant recruitment and study implementation. Written informed consent was obtained from all study participants and they received a copy in their preferred language (English, Hindi, Punjabi, or Urdu). There were no exclusion criteria.

### Outcome measures

The feasibility outcomes (operational, technical, resource, legal, and acceptability) of the study assessed whether the key components of CP@clinic could be implemented by paramedics with the help of volunteers in a South Asian community setting. Operational process measures included the total number of sessions held, the number of student volunteers, completeness of the data collected and missing data. Technical process measures were evaluated as the technical capability of the paramedic service to implement the program. Resource process measures were evaluated as the ability of the paramedic service to provide staffing. Legal process outcomes were the ability of paramedic service to conduct the program with the required oversight. Acceptability of the program was determined by the number of participants attending the sessions, and the number of participants attending more than one session, in addition to feedback through a key informant interview of a lead program volunteer. Health outcomes of all participants were assessed using two main categories of measures: risk factors for cardiometabolic disease and physical assessments (height, weight, body mass index [BMI], waist circumference, and BP). Waist circumference was collected using a measuring tape placed at the naval level. We attempted to collect objective waist measurements if possible, but if the individual refused for personal or cultural reasons, their pant size was requested. Weight was collected using a digital weighing scale, while height was self-reported. BMI was calculated using weight and height as kilograms per meter squared. BP was measured with an automated WatchBP Office BP device, a clinically validated machine that takes accurate BP measurements using an average of three readings [13]. Participants were classified as “hypertensive” if systolic BP was equal to or over 140 mmHg or diastolic BP was equal to or over 90 mmHg. Participants were categorized as having a “normal” BP if their reading was below 140/90. Similarly, BMI was categorized into three categories defined as “normal” (BMI < 23), “overweight” (23 ≤ BMI < 27.5) and “obese” (BMI ≥27.5) for South Asians [14].

Paramedics also assessed participants’ risk of type 2 diabetes using the Canadian Diabetes Risk Questionnaire (CANRISK) [15]. The students had specific training based on the available translation of CANRISK in Punjabi and were aware of culturally appropriate language to use that would convey the correct meaning. Based on their CANRISK scores, individuals were placed in three risk categories defined as 1) Low (CANRISK < 21), 2) Moderate (CANRISK 21–32), and 3) High (CANRISK > 32) based on the standard scoring algorithms. Participants with a moderate-to-high CANRISK were asked to return for a fasting capillary blood glucose test. Other health risk assessments included self-reported health history (heart disease history, stroke history, hypertension, high cholesterol, diabetes, number of medications), dietary intake (fruit and vegetable intake, salt intake), physical activity, smoking status, alcohol consumption, self-reported health status, health-related quality of life (HRQoL) based on the EQ-5D-3L [16], and whether the participant had a family physician.

### Data collection

Quantitative feasibility and process data were collected using document review of the CP@clinic program records and qualitative data were collected by conducting a key informant interview with one of the lead student volunteers who helped with the implementation of the sessions. The interview guide developed for this study is available as a supplemental file (see Additional file 1). Secondary outcomes of the study were collected by paramedics during the sessions using the CP@clinic database.

### Statistical analysis

Descriptive statistics summarized the process and feasibility measures of the study, sociodemographic characteristics, health behaviours and health outcomes of participants. To account for small numbers in some response options, the variables of self-reported heart disease history, stroke history, hypertension, high cholesterol, diabetes and taking medication for BP were collapsed into one variable called “indicator of cardiometabolic disease” if any of these were present. All analyses were performed using IBM SPSS Statistics 20.0. Qualitative data from the key informant interview was summarized, using thematic analysis, to supplement the information obtained from the program data.

## Results

### Process, feasibility, and challenges

#### Operational measures

The program was held weekly or bi-weekly in some months dependent on volunteer availability. Sessions were cancelled during major holidays or due to inclement weather. A total of 9 trained student volunteers assisted with translation and implementation of the CP@clinic sessions. Over the study period of 14 months, a total of 26 sessions were held (7 sessions at the recreation centre and 19 sessions at the local temple).

#### Technical measures

The paramedic service was able to leverage existing laptops used for their standard operations, on which the CP@clinic database was installed and therefore used to implement the CP@clinic program. The database did not require an active internet connection to function and could be synced after returning to their paramedic base station. Since the laptops were already used for recording personal health information during emergency calls, they were equipped with the appropriate encryption and other safeguards needed to securely store the CP@clinic program data.

#### Resource measures

The paramedic service had a small fleet of community paramedics who were available to staff the CP@clinic program for a half-day per week, without impacting acute services. This resource capacity enabled the program to be implemented without repercussions from local city council, since community paramedics were seen to be acting within their defined role of improving community health and preventing emergency calls.

#### Legal measures

Paramedics were able to perform the health risk assessments as part of their scope of practice with oversight provided from their base hospital. None of the assessments involved procedures or practices that were unfamiliar or required invasive measures. The grouping of the questions and tests that formed the health assessments collectively were new to the paramedics, but this was not significant enough to require new medical oversight. Decision support algorithms supplied to the paramedics through the CP@clinic database were based on already validated tools and evidence from the literature, and therefore were acceptable for this model of care.

#### Acceptability

The number of unique participants attending CP@clinic in the initial venue (recreation centre) was low (*n* = 19) despite implementing measures to increase recruitment. After 3 visits with no new participants, the venue was relocated to the local temple where participation was higher (*n* = 52). A total of 71 participants attended the CP@clinic sessions at the recreation center or the local temple. Among the 71 participants, 22 (31%) attended more than one CP@clinic session. There were an average of 5 participants per session with a range of 2–16 participants per session. For some weeks, the paramedics had an overwhelming number of attendees, demonstrating that the program was acceptable to this population and in this setting.

When the paramedic and volunteers were overwhelmed with the number of participants, only demographic variables and physical measures were obtained. They were unable to assist participants to complete the risk assessment questionnaires in the database. This occurred during the transition from the recreation centre to the temple. Furthermore there were occasions when volunteers had to cancel because of unavoidable circumstances. Therefore the paramedic, who could not speak any South Asian languages, could only take physical measures from participants because of the limited communication in English. This led to missing data in the CP@clinic database. However, on occasions when both paramedic and volunteers were present, the data collected was validated. Out of 28 completed data collection forms, we identified two cases where there were systematic data entry errors. Based on our audit, we found that either volunteer or paramedic missed sections of the risk assessment questionnaires in the database. We attribute this to user error which suggests the need for reminders or a refresher training program.

Qualitative results from the key informant interview identified that the committee members at the local temple had been welcoming and allowed the volunteers and paramedics to run the sessions in the common library. Holding the sessions on the weekends when people attended religious ceremonies at the temple was beneficial in recruiting study participants since these religious ceremonies are already well-attended. Some of the challenges faced by paramedics while recording physical measures and conducting risk assessments included language barriers, cultural appropriateness (male-female interactions) and the duration of the sessions. The volunteers helped overcome some of these challenges by translating the information and conducting risk assessments where appropriate. Four of the volunteers were females and could have assisted in waist circumferences measurements of female participants. The lead volunteer also highlighted that having more than one paramedic per session or increasing the duration of the sessions to 4 h, would have improved participant flow during busy hours since there was a high demand and many interested in the program. As a consequence, in some instances there was not enough time for all attendees to complete the full assessment, therefore demographic information and BP were taken, either solely or with a combination of some of the other assessments. Finally, the lead volunteer provided an insight on volunteers’ perspectives and experiences in the program and described the experience as “*a learning opportunity which made me aware of various health impacts of implementing a culturally-sensitive health promotion initiative.*”

### Demographic characteristics of participants

Demographic characteristics of the 71 study participants are listed in Table 1. The mean age of the study participants was 61.63 years (standard deviation [SD] = 11.14), ranging from 33 and 83. The majority of participants were male (56.3%) and were married (71.8%); 14.1% of participants had some high school education or less and 14.1% had a university or college degree; 85.9% of the total number of participants identified their mothers as South Asians and 87.3% identified their fathers as South Asians.

**Table 1:** Demographic characteristics of all study participants (*n* = 71) and the subset with risk factor assessments (*n* = 26)

Variable	All Study Participants Frequency ***n*** = 71 (%)	Subset of Participants Frequency ***n*** = 26 (%)
Gender		
Male	40 (56.3)	22 (84.6)
Female	11 (15.5)	4 (15.4)
Missing	20 (28.2)	0
Age		
Mean (SD)^a^	61.63 (11.1)	60.86 (11.0)
Missing	4 (5.6)	1 (3.8)
Marital Status		
Married	51 (71.8)	22 (84.6)
Separated	1 (1.4)	0
Single, never married	1 (1.4)	1 (3.8)
Widowed	3 (4.2)	1 (3.8)
Missing	15 (21.1)	2 (7.7)
Education		
Some high school or less	10 (14.1)	10 (38.5)
High school diploma	5 (7.0)	5 (19.2)
Some college or university	1 (1.4)	1 (3.8)
University or college degree	10 (14.1)	10 (38.5)
Missing	45 (63.4)	0
Ethnicity of Mother		
South Asian (East Indian, Pakistani, Sri Lankan, etc.)	61 (85.9)	24 (92.3)
East Asian (Chinese, Vietnamese, Filipino, Korean, etc.)	1 (1.4)	0
Other (Latin American, Arab, West Asian)	1 (1.4)	1 (3.8)
Missing	8 (11.3)	1 (3.8)
Ethnicity of Father		
South Asian (East Indian, Pakistani, Sri Lankan, etc.)	62 (87.3)	24 (92.3)
East Asian (Chinese, Vietnamese, Filipino, Korean, etc.)	0	0
Other (Latin American, Arab, West Asian)	1 (1.4)	1 (3.8)
Missing	8 (11.3)	1 (3.8)
Languages Spoken		
Arabic	1 (1.4)	1 (3.8)
Hindi	1 (1.4)	1 (3.8)
Punjabi	12 (16.9)	12 (46.2)
Urdu	2 (2.8)	2 (7.7)
Missing	55 (77.5)	10 (38.5)

^a^*SD* Standard deviation

Based on the study protocol, the paramedics had intended to obtain sociodemographic and health risk information from all study participants. However, it was only feasible to obtain complete health behaviours and risk factor information from a subset of 26 participants attending the sessions. For participants with complete risk factor assessment, their information was successfully faxed to their family doctors with their consent. Demographic characteristics of the subset of participants are also listed in Table 1.

Characteristics including health behaviours, risk factors, self-reported medical history, and quality of life, are reported for the subgroup in Table 2. In the subgroup, 80.8% were non-smokers and 76.9% did not consume alcohol. A large number of participants (76.9%) consumed fruits and vegetables every day and 65.4% performed some physical activity every day. Conversely, about 80% ate fast food once a week or more and 50% added salt to their food often or always. For self-reported health status and HRQoL, the majority of participants (88.5%) reported good, very good or excellent health status and no problems with mobility, self-care, usual care, pain and anxiety. In addition, 77% were overweight or obese, 42.3% had a moderate or high risk of diabetes based on their CANRISK score, and 65.4% had an indicator of cardiometabolic disease. Depending on their risk factors, health education and referrals were made to culturally appropriate community resources to help the participants obtain more information, make lifestyle changes, and improve their health.

**Table 2:** Health behaviours, risk factors and quality of life for the participants in the subgroup (*n* = 26)

Variable	Frequency n(%)
**Health Behaviours**	
Smoking Status	
Currently Smokes	2 (7.7)
Previous Smoker	3 (11.5)
Never Smoked	21 (80.8)
Alcohol Drinker	
No	20 (76.9)
Yes	5 (19.2)
Missing	1 (3.8)
Binge Drinking	
Never or less than once a month	24 (92.3)
More than once a week	1 (3.8)
Once a month	1 (3.8)
Alcohol Per Week	
1–5	5 (19.2)
6–10	1 (3.8)
11–15	1 (3.8)
Non-drinker/rare/have stopped drinking	19 (73.1)
Eats Fast Food	
0 meals	5 (19.2)
1–2 times per week	11 (42.3)
3 or more times per week	10 (38.5)
Stressed	
Rarely	17 (65.4)
Sometimes	7 (26.9)
Often	2 (7.7)
Salt Intake	
Never	2 (7.7)
Rarely	5 (19.2)
Sometimes	6 (23.1)
Often	8 (30.8)
Always	5 (19.2)
Lives Alone	
No	21 (80.8)
Yes	5 (19.2)
Physical Activity Daily	
No	9 (34.6)
Yes	17 (65.4)
Fruit and Vegetable Intake	
Less than once a week	0
Once a week	1 (3.8)
2–3 times per week	3 (11.5)
4–5 times per week	2 (7.7)
Everyday	20 (76.9)
**Risk Factors**	
Blood Pressure (BP)	
Normal	16 (61.5)
Elevated	10 (38.5)
Body Mass Index (BMI)	
Normal	6 (23.1)
Overweight	10 (38.5)
Obese	10 (38.5)
CANRISK Score^a^ (Risk of Diabetes)	
Low (< 21)	5 (19.2)
Moderate (21–32)	7 (26.9)
High (> 32)	4 (15.4)
Indicator of Cardiometabolic Disease	
No	9 (34.6)
Yes	17 (65.4)
**Self-reported Health Status**	
General Health	
Poor/Fair	3 (11.5)
Good	13 (50.0)
Very Good	6 (23.1)
Excellent	4 (15.4)
**Health Related Quality of Life**	
Mobility	
I have no problems in walking about	15 (57.7)
I have some problems in walking about	2 (7.7)
Missing	9 (34.6)
Self-care	
I have no problems with self-care	16 (61.5)
I have some problems with washing or dressing myself	1 (3.8)
Missing	9 (34.6)
Usual Activities	
I have no problems with performing my usual activities	16 (61.5)
I have some problems with performing my usual activities	1 (3.8)
Missing	9 (34.6)
Pain/Discomfort	
I have no pain or discomfort	11 (42.3)
I have moderate pain or discomfort	6 (23.1)
Missing	9 (34.6)
Anxiety/Depression	
I am not anxious or depressed	13 (50.0)
I am moderately anxious or depressed	4 (15.4)
Missing	9 (34.6)

^a^ This excludes participants who reported diagnosis of Diabetes (*n* = 10)

**Fig. 1:**
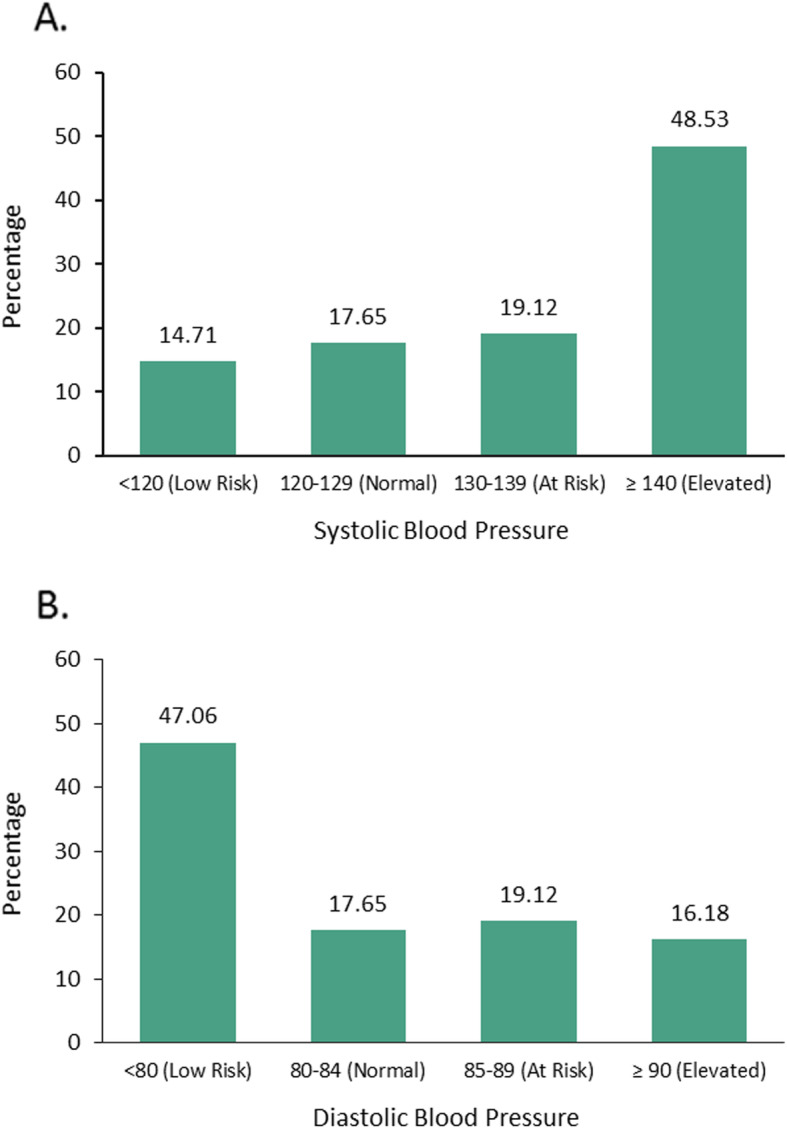
**a** Systolic blood pressure readings and **b** diastolic blood pressure readings for all study participants (*n* = 71)

BP readings were obtained for all study participants. The mean systolic BP was 138.74 (SD = 16.97) and the mean diastolic BP was 80.71 (SD = 10.49). Of all 71 participants, 46.5% had an elevated BP (≥140/90). See Fig. 1 for the distributions of participants across the SBP and DBP risk categories.

## Discussion

This feasibility study showed that the key components of CP@clinic can feasibly be implemented in a South Asian community setting. Implementing health-related programming in a culturally comfortable setting may offer a feasible approach to identifying individuals at high risk of developing cardiometabolic conditions. Our study was one of the first to assess the feasibility of using community paramedics and volunteers to implement a culturally-salient, evidence-based health promotion and disease prevention initiative, in a religious setting (a local Sikh temple in Hamilton, Ontario) to improve cardiometabolic risk factors among South Asians in Canada. We were able to recruit a total of 71 participants in this setting using student volunteers. A similar feasibility study of a cardiovascular screening program in a South Asian population in Alberta, Canada stated that places of worship play a strong social and cultural role in South Asian communities and are therefore, becoming increasingly popular settings within which to conduct health promotion initiatives [6]. Their study, similar to ours, concluded that implementing a volunteer-led health promotion program in religious settings was feasible.

There were no technical or legal challenges experienced in implementing CP@clinic with this population and setting, and paramedic services already had community paramedic staff available, although more paramedic staff resources would have been beneficial. Additionally, we demonstrated the acceptability of the program at a culturally sensitive facility through steady attendance rates, repeat attendance, and the increase in enrollment at the sessions held in the local temple, in comparison to the recreation centre. Moving the sessions from the recreation centre to the local Sikh Temple led to a substantial increase in the number of participants attending (*n* = 19 versus *n* = 52, respectively). Fewer individuals from this population used the recreation centre as compared to visiting the local temple and therefore, it was not a convenient location to hold the sessions. The lead student volunteer supported this view and identified the temple setting as being more convenient, educational and supportive of the CP@clinic program. Furthermore, the location of the program at the local temple allowed good access to a large proportion of this high-risk population who were visiting from local areas surrounding in Hamilton as well as the Greater Toronto Area (GTA), to attend religious ceremonies. There was a clear demand for the program as staff were, on occasions, overwhelmed with the number of participants attending the program which suggests the need for more volunteers or paramedics staffing the program. A qualitative study in London evaluated the National Health Services (NHS) cardiovascular health assessment program (NHS Health Checks) in religious settings, including South Asian temples. Their study found that participants found that advantages of implementing in religious and community settings included accessibility and community encouragement [17]. Therefore, our study findings and others in the literature suggests that religious institutions such as temples may provide valuable opportunities for health promotion and disease prevention initiatives to target specific high-risk communities.

Our study found that 46.5% participants had elevated BP compared to the hypertension prevalence of 23% among Canadians aged 20 to 79 years [18]. However it should be noted that our results are only from a small convenience sample of our study population since this is a feasibility study. Among the 26 participants in the subgroup, 42.3% had a moderate or high risk of developing diabetes based on their CANRISK score and 65.4% had an indicator of cardiometabolic disease. Although exact comparisons cannot be made to national data due to lack of available data and differing sampling methods, a recent study on Canadian South Asians aged 18 to 78, identified 16.1% of the total participants having a moderate or high risk of developing diabetes based on their CANRISK score [19]. This is much lower than the 42.3% observed in the current study. South Asians living in Canada have a higher prevalence and burden of diabetes and other cardiometabolic conditions compared to other ethnic groups [3]. A feasibility of the NHS Health Checks in religious South Asian settings in the UK found that their program was feasible and at the same time disclosed a similar pattern of elevated cardiometabolic risks [20] revealed by our study. Current literature have strongly encouraged health promotion initiatives and intervention strategies to reduce cardiometabolic risk factors among this high-risk group [1, 5].

The findings of our study, though only from a small sample, are consistent with other literature that shows there is a considerable burden of cardiometabolic risk factors in a South Asian community, supporting the need for future public health efforts to reduce their risk. However, these should be explored in a full scale study, with a larger sample, in wider and more varied settings that include different South Asian subgroups. The increased incidence of diabetes in a South Asian population can be attributed to several factors such as ethnic predisposition and lifestyle factors including dietary patterns and inadequate physical activity [21]. Dietary practices among South Asians often differ based on the form of religion practiced [22]. For example, many Hindus are vegetarians and consume a diet rich in carbohydrates and poor in protein. In comparison, Muslims often consume meat and the potential for a higher fat intake increases their risk of obesity and other cardiovascular diseases [22]. In addition, food preferences and health behaviors may also vary depending on the region of origin [22, 23]. For example, Punjabi (North Indian) cuisine is famous for being rich in calories due to generous use of cream and butter, which makes North Indians more susceptible to developing heart disease and diabetes [22]. Altogether, this study suggests that future research should be conducted to develop health promotion initiatives tailored to these diverse subpopulations within the South Asian community that could be implemented within a religious setting. Our study was unique in that it demonstrated the potential feasibility of using trained student volunteers as an option for a public health intervention to implement a community-based health assessment program in a South Asian community with a high proportion of recent immigrants. It should be noted that the involvement of volunteers in many countries seems to be increasing [24] and therefore research involving volunteers is definitely needed. Furthermore, our program would not have been possible without volunteers as all of our community paramedics were Caucasian and our target population of older adults struggled with English. Therefore this novel feasibility study offered a way to showcase bringing this very needed community paramedic resource to a population in need of it, supporting equitable access. The volunteers played a crucial role in facilitating communication between the paramedics and the participants by helping with translation. They also helped the paramedics in conducting risk assessments and guiding participants to appropriate community resources, when needed. During the key informant interview, the lead student volunteer stated that being involved in the program was beneficial for students as it helped enhance their knowledge and understanding of community-based program planning and implementation.

However, one of the challenges to involve student volunteers was their availability and commitment to the program. Some of the possible reasons for the lack of commitment and inconsistent volunteer participation may include school work load, family responsibilities, and lack of interest or experience. Since the paramedics relied on student volunteers for translation, they were unable to collect complete data for all the participants. Therefore, this study suggests that a combination of student volunteers and a paid professional translator, or implementation by a health professional from the community who can speak the languages, may be required for successful dissemination of future health promotion initiatives conducted in a similar context.

There are some potential limitations of this feasibility study. First, the study participants were primarily North Indian and therefore, the findings may not be generalizable to other South Asian groups or religious settings. The study participants may also differ from the general South Asian population in terms of a variety of other factors, such as the priorities of the religious congregation, demographics, health concerns and interests. Thus, further formative research is essential to design and implement health promotion and disease prevention programs tailored to address specific interests and concerns of various subgroups within a community. Secondly, this study had a relatively small sample size and a lack of complete data for all participants. Though 63% of data were missing data for the whole assessment, we believe reporting our results was necessary to report the challenges of conducting such a program in the South Asian setting. Our program was successfully implemented but we learned that there were many issues that need to be considered before going into a full scale study, such as the high demand for an intervention in this setting, which requires additional paramedics and volunteers. Consequently, collecting follow-up data was unfeasible, which may also limit the generalizability of the study results to the larger general population. Since little is known about the impact of community-based health promotion interventions in religious settings within Canada, it is suggested that future research with this high-risk ethnic group should be conducted with a larger sample, and in different religious and cultural settings to effectively understand the potential impact of programs such as CP@clinic on the health outcomes in this population. Lastly there are some limitations in our feasibility assessment in that we were not able to assess. These were mainly the acceptability of the program from the paramedics’ perspective, from the Temple Management perspective and from the primary care providers perspective.

## Conclusion

In conclusion, the findings of this study suggest that implementing the key components of CP@clinic as a culturally sensitive community-based health assessment program in a South Asian religious setting is feasible. There is a clear demand for the program. However, there is a need to increase staffing, improve volunteer retention and commitment, and assure complete data collection. Once these hurdles have been managed, it is imperative to evaluate the impact of this study on a larger scale to determine the effectiveness of the CP@clinic program in decreasing cardiometabolic risk factors and health outcomes. Overall, this study suggests that CP@clinic was feasible and welcomed in the South Asian place of worship setting, and additionally that there is a high proportion of cardiometabolic risk factors among attending participants.

## Supplementary Information

**Additional file 1.** Key informant interview guide. This guide was used to interview the lead volunteer of the program to assess his perceptions about the CP@clinic program and how to improve it.

## Data Availability

The data that support the findings of this study are not publicly available due to them containing information that could compromise participant privacy. De-identified, limited data will be shared by the lead author upon request.

## Abbreviations

BMI: Body mass index
BP: Blood pressure
CANRISK: Canadian Diabetes Risk Questionnaire
CP@clinic: Community Paramedicine at Clinic
HRQoL: Health-related quality of life
SD: Standard deviation
