# Supplementary material for: Feasibility of implementing a community cardiovascular health promotion program with paramedics and volunteers in a South Asian population
Source: BMC Public Health. 2020 Oct 27;20:1618. doi: 10.1186/s12889-020-09728-9 (PMC7590723; doi:10.1186/s12889-020-09728-9)
Supplement: Supplementary file 1 — Additional file 1. Key informant interview guide. This guide was used to interview the lead volunteer of the program to assess his perceptions about the CP@clinic program and how to improve it. [file 12889_2020_9728_MOESM1_ESM.docx]

**Additional File 1:** Key Informant Interview (KII) Guide

**Project Title:** A Community Paramedicine Initiative for Older Adults Living in Subsidized Housing: Expanding to Other Communities

**Description:** These KII questions were intended for student volunteers of the CP@clinic expansion to the South Asian communities. This study has been reviewed by the Hamilton Integrated Research Ethics Board (HIREB). The HIREB is responsible for ensuring that participants are informed of the risks associated with the research, and that participants are free to decide if participation is right for them. If you have any questions about your rights as a research participant, please call the Office of the Chair, Hamilton Integrated Research Ethics Board at 905.521.2100 x 42013.

1. Can you tell me about your experience/opinions about participating in the Riverdale study?
2. What was your role in this study?
3. For how long did you participate in this study?
4. Can you tell me more about the study setting, i.e. community centre and the Sikh temple? Were there any challenges specific to these locations?
5. How long were the CP@clinic sessions?
6. How often were the sessions held?
7. How were participants recruited in the study?
8. How was the participation rate? Any difference in the participation rate at the community centre and the temple?
9. Did you come across any challenges during your participation in this study?
10. Are you aware of any challenges experienced by the study participants?
11. Are you aware of any challenges experienced by the paramedics?
12. What did you like the most about participating in this study? Any take-aways from this experience?
13. Do you have any suggestions on how we can make this a better experience for the volunteers in the future?
14. Do you have any other comments/feedback based on your participation in this study.
